# 2,2-Diphenyl-1-picrylhydrazyl as a screening tool for recombinant monoterpene biosynthesis

**DOI:** 10.1186/1475-2859-12-76

**Published:** 2013-08-23

**Authors:** James BYH Behrendorff, Claudia E Vickers, Panagiotis Chrysanthopoulos, Lars K Nielsen

**Affiliations:** 1 Australian Institute for Bioengineering and Nanotechnology, The University of Queensland, St Lucia QLD 4072, Australia; 2 Metabolomics Australia (Queensland Node), The University of Queensland, St Lucia QLD 4072, Australia

**Keywords:** Monoterpene, Limonene, Biosynthesis, Screening, 2,2-diphenyl-1-picrylhydrazyl, DPPH, Antioxidant, *Saccharomyces cerevisiae*, Isoprenoid

## Abstract

**Background:**

Monoterpenes are a class of natural C_10_ compounds with a range of potential applications including use as fuel additives, fragrances, and chemical feedstocks. Biosynthesis of monoterpenes in heterologous systems is yet to reach commercially-viable levels, and therefore is the subject of strain engineering and fermentation optimization studies. Detection of monoterpenes typically relies on gas chromatography/mass spectrometry; this represents a significant analytical bottleneck which limits the potential to analyse combinatorial sets of conditions. To address this, we developed a high-throughput method for pre-screening monoterpene biosynthesis.

**Results:**

An optimised DPPH assay was developed for detecting monoterpenes from two-phase microbial cultures using dodecane as the extraction solvent. The assay was useful for reproducible qualitative ranking of monoterpene concentrations, and detected standard preparations of myrcene and γ-terpinene dissolved in dodecane at concentrations as low as 10 and 15 μM, respectively, and limonene as low as 200 μM. The assay could not be used quantitatively due to technical difficulties in capturing the initial reaction rate in a multi-well plate and the presence of minor DPPH-reactive contaminants. Initially, limonene biosynthesis in *Saccharomyces cerevisiae* was tested using two different limonene synthase enzymes and three medium compositions. The assay indicated that limonene biosynthesis was enhanced in a supplemented YP medium and that the *Citrus limon* limonene synthase (CLLS) was more effective than the *Mentha spicata* limonene synthase (MSLS). GC-MS analysis revealed that the DPPH assay had correctly identified the best limonene synthase (CLLS) and culture medium (supplemented YP medium). Because only traces of limonene were detected in SD medium, we subsequently identified medium components that improved limonene production and developed a defined medium based on these findings. The best limonene titres obtained were 1.48 ± 0.22 mg limonene per L in supplemented YP medium and 0.9 ± 0.15 mg limonene per L in a pH-adjusted supplemented SD medium.

**Conclusions:**

The DPPH assay is useful for detecting biosynthesis of limonene. Although the assay cannot be used quantitatively, it proved successful in ranking limonene production conditions qualitatively and thus is suitable as a first-tier screen. The DPPH assay will likely be applicable in detecting biosynthesis of several other monoterpenes and for screening libraries of monoterpene-producing strains.

## Background

Monoterpenes are a class of naturally-occurring C_10_ compounds with many potential high-value applications including use as biofuels, feedstocks for pharmaceutical and other industrial product syntheses, and flavours and fragrances [1-5]. These compounds are derived from the polymerization of two C_5_ isoprenoid monomers, isopentenyl diphosphate and dimethylallyl diphosphate, to form geranyl diphosphate (GPP). The C_10_ GPP is then subject to rearrangements by different monoterpene synthase enzymes to produce the array of monoterpene compounds found in nature [6,7]. Due to the difficulty in extracting commercially-viable quantities of monoterpenes from native sources, there is an increasing interest in engineering industrial microorganisms for biosynthesis of these compounds [8-11].

The yield and purity of monoterpenes are typically analysed *via* gas chromatography–mass spectrometry (GC-MS). In a strain engineering context it is often necessary to test numerous mutant strains and fermentation conditions for improvements in product yield. Furthermore, many modifications may in fact not result in monoterpene production. Analysis of combinatorial sets of strains and fermentation conditions with GC-MS represents a severe bottleneck in the engineering workflow. A high-throughput method for screening micro-encapsulated *Saccharomyces cerevisiae* that produce water-immiscible isoprenoid compounds was recently developed [12], but the microfluidics and fluorescence-activated cell sorting infrastructure required for this method is not available to many laboratories. A technically simple and rapid pre-screening method for identifying fermentation conditions and gene combinations that result in monoterpene biosynthesis would facilitate a reduction in the number of samples that need to be analysed with GC-MS and would greatly expedite monoterpene metabolic engineering efforts.

**Figure 1:**
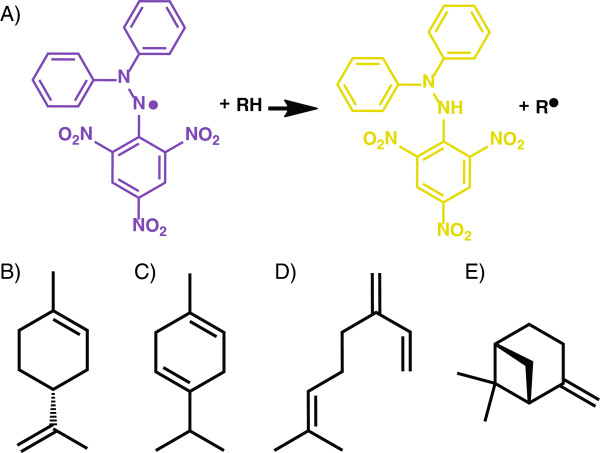
**The DPPH reaction and monoterpene compounds examined in this study.** DPPH exhibits strong absorbance at 517 nm (purple) which decreases proportionately with the loss of its radical **(A)**. The chemical structures of monoterpene compounds (+)-limonene **(B)**, γ-terpinene **(C)**, myrcene **(D)**, and β-pinene **(E)** are shown.

2,2-Diphenyl-1-picrylhydrazyl (DPPH) is a stable radical that exhibits strong absorbance at 517 nm. The absorbance at 517 nm decreases proportionately with the loss of the radical in exchange for a proton, resulting in a colour change from purple to yellow (Figure 1A). DPPH can be used to accurately titrate the oxidisable groups of biomolecules [13], and has commonly been used to estimate the antioxidant capacity of complex mixtures including plant oils, many of which contain high concentrations of monoterpenes [14-17]. We therefore thought that DPPH might be used as a screening tool for monoterpene biosynthesis by industrial microorganisms. DPPH assays reported in the literature to-date are unsuitable for this purpose as they are usually optimised for use with solvents that are incompatible with microbial growth, and detection limits for different individual monoterpenes have not been determined.

We present here a modified DPPH radical-scavenging assay that enables its use as a tool for identifying the best monoterpene-producing cultures from a set. We assessed the potential for using this assay in the detection of limonene and β-pinene (proposed jet fuel substitutes), myrcene (a renewable chemical feedstock), and γ-terpinene (a proposed jet fuel substitute that is also used in semiconductor manufacture) (Figure 1B-E). We applied the assay to identify appropriate genes and culture media for the production of limonene in *S. cerevisiae*, and then used this information to develop an improved defined medium for limonene biosynthesis. Assay conditions were optimised for use in microtitre plates with dodecane as the solvent for both DPPH and the monoterpene samples, as dodecane has been established as a suitable non-toxic solvent for the recovery of hydrophobic compounds from live microbial cultures [18,19].

## Results and discussion

### Optimization of assay conditions

Dodecane is a preferred solvent for the extraction of hydrophobic compounds from live cultures due to its low toxicity and good phase separation [20-22]. We optimized the DPPH assay for use with dodecane, as previously-published DPPH radical-scavenging assays used methanol or ethanol as the solvent [23]. The maximum absorbance of DPPH dissolved in dodecane was 510 nm (Additional file 1A), similar to the previously-published value of 517 nm for DPPH dissolved in methanol [23]. The peak was relatively broad, and wavelengths immediately above and below this maximum might also be suitable for use. A standard curve of DPPH in dodecane demonstrated a linear relationship (*R*^2^ = 0.999) between DPPH concentration and A_510 nm_ up to 1 mM DPPH (Additional file 1B). Based on these results, subsequent experiments used DPPH at a final concentration of 100 μM and absorbance was monitored at 510 nm.

A slow decrease in A_510 nm_ was observed when DPPH was dissolved in dodecane. Minimizing the background reaction rate was important for the detection of low concentrations of monoterpenes. Microtitre plate composition and the effect of dissolved gases were investigated. When the reaction rates of positive and negative controls were compared, a greater difference, and therefore greater sensitivity, was observed when polypropylene microtitre plates were used instead of standard polystyrene plates (Additional file 1C). The reduced sensitivity observed with polystyrene reaction vessels may be due to a weak reaction between DPPH and polystyrene [24,25]. It has previously been reported that polypropylene microplates are better suited to handling hydrophobic compounds than polystyrene and are resistant to degradation by a broader range of chemicals [26,27]. No significant difference in reaction rate was observed between samples where dodecane was treated with nitrogen or air prior to dissolving DPPH, indicating that dissolved oxygen was not a significant contributor to the background reaction rate (Additional file 1D). DPPH reacted more quickly with fresh dodecane than with dodecane that had been incubated with *S. cerevisiae* culture, but this difference was not statistically significant (Additional file 1E). Importantly, this showed that DPPH-reactive compounds do not accumulate to detectable levels in the dodecane phase when dodecane is incubated with *S. cerevisiae* EPY210C carrying the empty expression vector. The bromine test indicated the presence of unsaturated compounds in fresh dodecane and certification was obtained from the supplier that the dodecane batch contained 99.6% dodecane. The presence of reactive unsaturated contaminants in dodecane may have contributed to the background reaction rate. The effect of reactive compounds on the reaction rate with DPPH is additive, so the background reaction only becomes problematic if a weakly-reactive compound is being examined or if a contaminant compound is very strongly-reactive. Therefore, this effect should be controlled for by using dodecane from a single source in each experiment and including appropriate negative and positive controls (i.e. assay of monoterpene standards).

### Hit-identification thresholds and quality of screening assay for various monoterpenes

**Figure 2:**
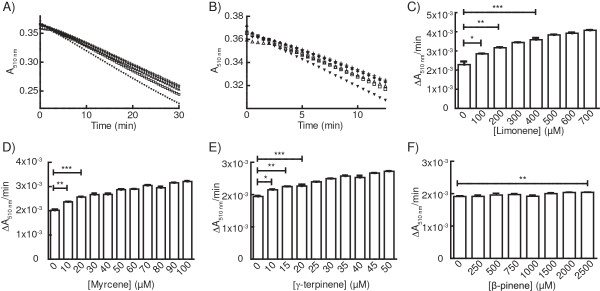
**Detection of monoterpenes by incubation with DPPH. (A)** Sample output are shown for 100 μM DPPH incubated with 0 μM (+), 100 μM (♦), 200 μM (Δ), 800 μM (□), and 1600 μM limonene (▼), with an expanded section of this data to 12.5 min shown in **(B)**. The rates of reaction between 100 μM DPPH and varying concentrations of limonene **(C)**, myrcene **(D)**, γ-terpinene **(E)**, and β-pinene **(F)** were calculated with a linear regression of the data collected in period of the reaction between 7–12 min (n = 3 for each concentration, mean ± 1 SD). Reaction rates for DPPH incubated with monoterpene standards were compared to the relevant negative controls using an unpaired Student’s *t*-test (* = p <0.05, ** = P <0.01, *** = p < 0.001).

A range of concentrations were tested for each monoterpene in order to determine the lowest concentration at which a difference in ΔA_510 nm_/min could be observed between samples containing standard preparations of monoterpene and negative controls (Figure 2). Typical output data for varying concentrations of limonene (0, 100, 200, 800, and 1600 μM) are included in Figure 2A as an example, with a close-up of the first 12.5 min shown in Figure 2B. Trials with myrcene and γ-terpinene gave similar responses at lower concentrations. Reaction rates for different substrate concentrations were compared to negative controls with an unpaired Student’s *t*-test. Given that we seek to reduce the number strains for second-tier screening *via* GC-MS analysis, we set a stringent threshold to reduce the occurrence of false positives, rather than a more relaxed threshold that would minimize false negatives. We defined the hit-identification threshold as a reaction rate that was significantly different to negative controls with p < 0.01 (Student’s *t* test, n = 3). According to this constraint, limonene was detected at 200 μM (Figure 2C), myrcene at 10 μM (Figure 2D) and γ-terpinene at 15 μM (Figure 2E). No significant difference in reaction rate between negative controls and β-pinene standards was observed at less than 2.5 mM β-pinene (Figure 2F). DPPH-scavenging activity was readily observed with β-pinene at concentrations > 10 mM (data not shown).

Recent monoterpene engineering studies reported titres of 1.7 mg pinene per L (12.5 μM) [28], and 56.8 mg limonene per L (416.9 μM) [10]. The reported pinene titre is below what would be detectable even if it were concentrated into a dodecane phase of 1/100th the culture volume, while the reported limonene concentration would certainly be above the detection limit when extracted with the 20% (v/v) dodecane overlay that the authors used in their study [10]. The DPPH assay is unlikely to be useful for detection of heterologous β-pinene biosynthesis but should be useful for the sensitive detection of myrcene, γ-terpinene and limonene (and probably some other monoterpenes not examined in this work). The relationship between chemical structure and reaction rate with DPPH is complex and several studies have attempted to elucidate structure-activity relationships. These studies have focused on flavonoids and other phenolic compounds, identifying the number, position and acidity of hydroxyl groups as important factors [29-33]. Although there is no simple trend in the present study, the reaction rate is likely influenced by the number and position of double bonds. As antioxidant compounds donate a proton to the DPPH radical [34], greater weighting may be given to double bond positions that increase the availability of allylic protons (due to the weaker C-H bond at allyl groups).

The assay was highly reproducible when analytical standards dissolved in dodecane were used. This indicates that when the assay is applied to culture extracts, variation observed between replicate samples is due to differences between fermentations rather than some technical aspect of the DPPH assay. Reactions containing different concentrations of monoterpenes could be ranked by calculating the slope of the curve where the reaction rate was linear, and also by directly observing the kinetic plot of the assay (e.g. Figure 2A). Ideally it would be possible to compare assay results directly to a standard curve, but caution should be exercised here as incubation of dodecane with live microbial cultures may affect the background reaction rate, preventing direct comparison to standards prepared with fresh dodecane. Appropriate pre-testing and controls should therefore always be included, and the reagents used in each experiment (particularly DPPH and dodecane) should be sourced from a single production batch in order to minimise variability (since the ratios of DPPH crystalline forms may be variable between batches and since dodecane may have different background rates between batches). Furthermore, it became more difficult to capture the true initial reaction rate as the concentration of monoterpene increased due to the time lag between reads in the microplate reader. Although different monoterpene concentrations could still be easily ranked simply by observing the raw data, the delay between reads prevented the construction of a linear standard curve other than across a narrow range of concentrations close to the hit identification threshold. In the event that highly reactive monoterpenes or high concentrations prevent the comparison of initial reaction rates, we propose that samples are simply diluted further in dodecane. Alternately, reactions that rapidly run to completion could be ranked according to T_50%_ (the time taken to deplete 50% of the initial concentration of DPPH). Comparison of T_50%_ values is an established method for ranking antioxidant capacities of complex mixtures [35].

### Screening for limonene biosynthesis in *S. cerevisiae*

**Figure 3:**
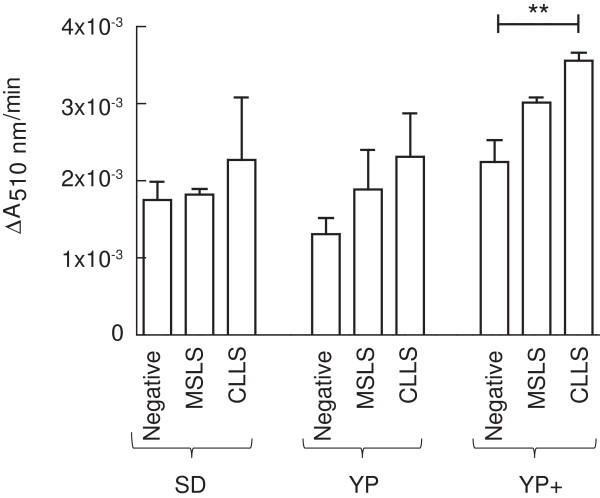
**Detection of limonene biosynthesis in a small-scale screen.***S. cerevisiae* EPY210C expressing the *C. limon* (CLLS) or *M. spicata* (MSLS) limonene synthase, or carrying an empty expression vector (Negative) were cultured for 120 h in 5 mL of either SD, YP, or YP + medium (n = 3 biological replicates in each condition). Reaction rates between dodecane extracts and DPPH (100 μM) were calculated and compared to the negative control for each medium composition (mean ± 1 SD, unpaired Student’s *t*-test, ** = p < 0.01).

*S. cerevisiae* EPY210C expressing limonene synthases from either *C. limon* (CLLS) or *M. spicata* (MSLS) were cultured in small-scale in SD, YP, or YP + media, and analysed for limonene production after 120 h using the DPPH assay (Figure 3). *S. cerevisiae* EPY210C carrying the empty expression vector was included as a negative control. In all medium compositions, samples from CLLS cultures reacted with DPPH at a greater rate than negative controls, but this difference was only statistically significant when YP + medium was used. The mean reaction rate of MSLS samples was slightly greater than negative controls when YP and YP + media were used. Reaction rates of negative control samples differed between the three media compositions, and some component of YP + medium or a metabolite produced as a consequence of growth in YP + medium may contribute to the background reaction rate and account for the greater reactivity observed in YP + samples. However, the greatest absolute difference in reaction rate between samples from CLLS cultures and negative controls was observed when grown in YP + medium, indicating that the use of YP + medium increased the production of limonene.

**Figure 4:**
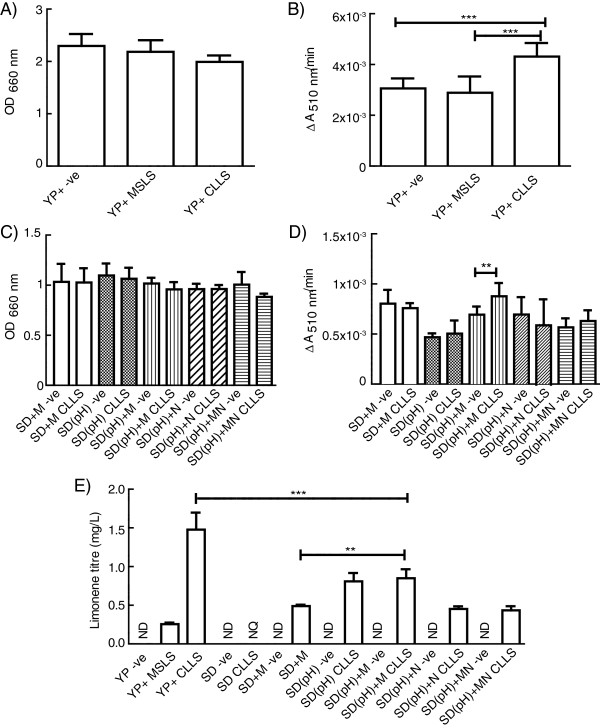
**Limonene biosynthesis in 50 mL cultures.***S. cerevisiae* EPY210C expressing the *C. limon* (CLLS) limonene synthase, *M. spicata* limonene synthase (MSLS), or carrying an empty expression vector (−ve) were cultured for 120 h in 50 mL of culture medium. Initially strains were cultured in supplemented YP medium (YP+) and the optical density **(A)** and reaction rate of the dodecane phase with DPPH **(B)** were recorded. This experiment was repeated with –ve and CLLS cultures in several defined medium compositions **(**panels **C** and **D)**: SD medium containing extra metals (SD + M), pH adjusted SD medium (SD(pH)), pH adjusted SD medium with extra metals (SD(pH) + M), extra nitrogen (SD(pH) + N), or extra metals and nitrogen (SD(pH) + MN), or supplemented YP medium (YP+). Limonene titres **(E)** were calculated following GC-MS analysis of dodecane extracts. All data shown are calculated from n = 3 biological replicates, showing mean ± 1 SD. ND = not detected, NQ = not quantifiable. Results were compared to negative controls using an unpaired Student’s *t*-test (** = p < 0.01, *** = p < 0.001).

On the basis of these data, YP + was selected as the culture medium for examining the two limonene synthase strains in 50 mL fermentations. After 120 h, the biomass was recorded by measuring OD_660_ (Figure 4A) and the dodecane phase was harvested. The reduction in biomass observed in limonene synthase cultures could be due in part to limonene toxicity [20], the burden of plasmid maintenance and/or the burden of limonene synthase protein overexpression in these strains. In large scale fermentations, dodecane samples from CLLS cultures reacted with DPPH significantly faster than the negative control or samples from MSLS cultures (Figure 4B). The dodecane phase was also analysed *via* GC-MS. Limonene, identified by comparison to retention time and characteristic ions of authentic standards, was detected in dodecane from both CLLS (542 ± 81 μM, n = 3 ± SD) and MSLS cultures (94 ± 7 μM, n = 3 ± SD) grown in YP + medium. Limonene was not detected in negative controls. These concentrations represent only the limonene that partitioned into the 1 mL dodecane phase from the 50 mL culture, and therefore suggest titres of at least 1.48 ± 0.22 mg limonene per L CLLS culture and 0.26 ± 0.02 mg limonene per L MSLS culture. In the DPPH assay the dodecane samples were diluted 1 in 2 (100 μL sample mixed with 100 μL DPPH). Therefore, in the DPPH assay CLLS samples contained ~270 μM limonene (which is slightly above the 200 μM detection limit of the assay defined in Figure 2C), while MSLS samples contained ~45-50 μM limonene (which is below the threshold for positive hits). Limonene production by CLLS in SD medium was also observed when analysed by GC-MS, but the limonene detected was below the lower limit of quantification. The calculated limonene titres for each condition trialled are shown in Figure 4E. These data demonstrate that more limonene was produced by *S. cerevisiae* EPY210C expressing the *C. limon* limonene synthase than by the same strain expressing the *M. spicata* limonene synthase, and that limonene production was enhanced in YP + medium, which is in agreement with the results of the DPPH assay.

Limonene biosynthesis *via* limonene synthase enzymes requires an intracellular supply of the substrate, GPP. No GPP synthase has been identified in *Saccharomyces* to-date, but biosynthesis of monoterpenes in non-engineered strains has been demonstrated previously under specific fermentation conditions [36]. In particular, biosynthesis of citronellol and linalool were stimulated under microaerobic conditions and when the concentration of assimilable nitrogen in the culture medium was increased [36]. Free GPP has been measured in wild-type *S. cerevisiae* strains [37] and may be produced as a by-product of the farnesyl diphosphate synthase, which proceeds through a GPP intermediate [38].

The use of a complex medium in a strain engineering context is disadvantageous as product yields cannot be calculated accurately and many metabolites cannot be quantified if the exact composition of the medium is not known. For this reason, we sought to use the data generated thus far to develop a defined medium that supported limonene biosynthesis by our strains. We identified pH, nitrogen content and trace metals as key differences between YP + and SD media which might be relevant for limonene production. The limonene synthase enzyme requires divalent cations (magnesium or manganese) for activity [39], and it is possible that metals are limiting in SD and YP media. Nitrogen has been shown to affect endogenous monoterpene production in *S. cerevisiae*[36]. We calculated that YP and YP + medium used in this experiment contained approximately 3.3 g nitrogen/L while SD medium contained 1.1 g nitrogen/L, suggesting that monoterpene biosynthesis could potentially be improved by increasing the nitrogen content of the medium. We prepared five different media to test the effects of these components (media compositions are compared in Additional file 2). These media were: complete SD medium plus 2 mM magnesium sulfate and trace metals (SD + M); SD medium adjusted to pH 6.3 (SD(pH)); SD medium pH 6.3 plus trace metals and 2 mM magnesium sulfate (SD(pH) + M); SD medium pH 6.3 supplemented with ammonium sulphate to contain the same total nitrogen as YP + medium (SD(pH) + N, see Methods); and SD medium pH 6.3 containing both the extra ammonium sulfate and extra trace metals and magnesium sulfate (SD(pH) + MN). Full details of the additives are described in the Materials and methods. YP and YP + media had an initial pH of 6.3, while unmodified SD medium had a pH of 4.55. The negative control strain and CLLS were grown in these media under the same conditions as for the 50 mL YP + medium fermentations with n = 3 biological replicates. Due to the likelihood that *S. cerevisiae* metabolism will differ between the various culture media trialled, CLLS strains were compared to negative control strains grown in the same medium and not to CLLS strains grown in other media. After 120 h, the biomass was recorded (Figure 4A) and aliquots of the dodecane phase were sampled for the DPPH assay.

All cultures grown in the supplemented SD media reached a similar cell density (Figure 4C), which was about half that observed for YP + medium (Figure 4A). In the DPPH screening assay, the greatest positive difference in reaction rate between CLLS extracts and negative controls was observed with SD(pH) + M medium (Figure 4D). Subsequent GC-MS analysis revealed that of the supplemented SD media, SD(pH) + M produced the most limonene (0.9 ± 0.15 mg limonene/L), followed by SD(pH) (0.81 ± 0.11 mg limonene/L), SD + M (0.49 ± 0.02 mg limonene/L), SD(pH) + N (0.45 ± 0.03 mg limonene/L) and SD(pH) + MN (0.43 ± 0.05 mg limonene/L) (Figure 4E).

While addition of trace metals + magnesium and adjusting the pH both produced significant increases in limonene, the effect was not cumulative, as limonene produced in SD(pH) and SD(pH) + M media were not significantly different. The influence of pH on limonene production may relate to the relatively narrow functional pH range of the limonene synthase [39,40]. The optimum pH for *C. limon* limonene synthase is 7.0 [39], and other limonene synthases have also been described with an optimal pH of 7.0 and half maximum velocity at approximately pH 6.0 [40,41], and minimal activity below pH 5.5 [40]. Although eukaryotic cells are adapted to regulate their intracellular pH, the extracellular pH (i.e. culture medium pH) does influence cytoplasmic pH in *S. cerevisiae* to an extent [42-45], with intracellular pH values between 5.3-5.7 generally observed when the extracellular pH was between 3–5.5 [42,43,45].

Addition of nitrogen did not improve limonene production; in fact, limonene production was decreased when nitrogen was added to SD(pH) and SD(pH) + M media. It is not clear why increased nitrogen content in defined medium suppressed limonene production. Although nitrogen content has been demonstrated to affect the production of linalool [36,46] and citronellol [36] by *S. cerevisiae*, the effect of increased nitrogen on productivity may be strain-specific [46], and the nitrogen concentrations tested were below that of unmodified SD medium. Furthermore, additional nitrogen in SD + N medium was added as ammonium sulfate, whereas in YP medium nitrogen is provided primarily by digestion of peptides. The role of ammonia as a regulator of gene expression in *Saccharomyces* is extremely complex (reviewed in [47]), and having such an excess of ammonium may be disadvantageous due to some unknown regulatory process. Alternately, excess ammonium may adversely affect limonene biosynthesis by changing the pH [48].

In all media tested the greatest reaction rate with DPPH was observed in extractions from CLLS cultures, but the response was only significantly different to negative controls when YP + medium was used. GC-MS analysis confirmed that the greatest titre of limonene was obtained when cultures were grown in YP + medium. The initial pH of the medium was an important factor in limonene production. All five modified SD media produced quantifiable limonene, whereas SD medium prepared according to the manufacturer’s instructions produced trace amounts of limonene below the limit of quantification. Limonene titres of approximately two-thirds that obtained in YP + medium were possible in a pH-adjusted SD medium supplemented with metals, but given the reduced cell density in SD medium the yield was similar on a cell density basis. Supplementation with additional metals improved production, but the starting pH of the medium appeared to be most important factor of those tested. Clearly, culture conditions (including medium composition) are an important contributor to recombinant monoterpene biosynthesis.

## Conclusion

Here we describe a qualitative assay for monoterpene biosynthesis in heterologous systems based on the rate of reaction between DPPH and monoterpenes produced from live cultures, using dodecane as an extractant. The sensitivity of the assay depends on the particular monoterpene of interest: for example, myrcene and γ-terpinene were detectable at concentrations as low as 10 μM and 15 μM, respectively, while limonene was classed as detectable at concentrations above 200 μM. Although the amount of limonene produced under our test conditions was below the 200 μM detection limit when calculated based on total culture volume, detection was possible using the DPPH assay because the limonene was concentrated into a reduced volume of dodecane during fermentation. We anticipate that the DPPH assay will be a useful complement to the increasing popularity of two-phase extraction from live cultures using dodecane [20]. The DPPH assay was useful for identifying the optimal culture medium for limonene biosynthesis out of those tested, and will likely be useful generically for determining optimal fermentation conditions. The assay also correctly identified which of two limonene synthases were the most effective in producing limonene (as confirmed by GC-MS analysis). Of the conditions tested, the greatest limonene production could be observed using the *C. limon* limonene synthase in a supplemented rich medium (YP+). However, we were able to develop an improved defined medium for limonene production which might be more suitable in an industrial setting. Our experiments suggest that the DPPH assay will be transferrable to also detecting monoterpenes other than limonene,and may be useful for screening large libraries or strains and fermentation conditions in instances where monoterpene production already meets the sensitivity threshold for the compound of interest.

## Materials and methods

### Chemicals

DPPH, dodecane (ReagentPlus grade), (*R*)-(+)-limonene, myrcene, (−)-β-pinene, and γ-terpinene were purchased from Sigma-Aldrich (St Louis, MO., USA). Synthetic dextrose (SD) medium components were purchased from MP Biomedicals (Santa Ana, CA., USA). Other chemicals and media components were of the highest quality locally available.

### Assay development

All DPPH solutions were prepared freshly in dodecane immediately prior to use. Spectroscopy was performed using a Spectramax M5 plate reader (Molecular Devices, CA., USA) at 25°C. Assay conditions were determined by confirming the spectral characteristics of DPPH dissolved in dodecane with an absorbance scan and standard curves. A slow reaction between DPPH and dodecane was observed, and possible causes of this reaction were investigated with the aim of minimising the assay background. The role of reaction vessel composition was investigated by comparing the reaction of limonene (500 μM) with DPPH (100 μM) in dodecane (total volume 200 μL) to negative controls (100 μM DPPH in 200 μL dodecane) in standard polystyrene 96-well plates (Greiner Bio-One cat. no. 655–101) and in polypropylene 96-well plates (Greiner Bio-One cat. no. 655–201, Greiner Bio-One, Belgium). The effect of dissolved gases was examined by comparing the background reaction rates of DPPH (100 μM) dissolved in dodecane that had either been aerated by vortex mixing for 10 min or bubbled with nitrogen gas for 10 min. Dodecane was tested for the presence of unsaturated contaminants using the bromine test for alkenes [49]. One drop of bromine was added from a Pasteur pipette to 1 mL dodecane and observed for 5 min, and compared to samples of ultrapure water treated likewise. We also tested whether the background reaction rate of DPPH in dodecane would be affected by incubation of dodecane with live cultures. Overnight starter cultures of *S. cerevisiae* EPY210C (described below in *Fermentation conditions*) were diluted 100-fold into 50 mL YPD medium [50] with 5 mL dodecane in screw-cap non-baffled shake flasks (n = 3 replicate cultures each). After 72 h incubation with shaking (200 rpm, 25 mm orbit) at 30°C, the dodecane layer was separated by centrifugation at 4000 × *g* for 10 min. Dodecane overlay samples (100 μL) were mixed with DPPH (100 μM in a final volume of 200 μL) and the reaction rate was compared to the reaction of DPPH in fresh dodecane.

### DPPH assay conditions and analysis

Monoterpenes dissolved in dodecane (100 μL, various concentrations) were added directly to 100 μL of 200 μM DPPH in polypropylene 96-well plates and mixed for 1 s using the auto-mix function of the plate reader. Preliminary experiments determined that mixing was essential, and poorly-mixed samples resulted in noisy recordings where increases in absorbance were observed as well as decreases. Given the viscous nature of dodecane, it is absolutely critical to this assay that samples are completely mixed without introducing excess oxygen or forming bubbles. We found that 1 s mixing time using the automix function of the plate reader resulted in reproducible reaction curves with limonene standards (Additional file 1F-G). Alternately, reproducible curves could be obtained by stirring with pipette tips when adding DPPH to the sample. Reactions were monitored for 30 min, recording the absorbance at 510 nm every 30 s. The sensitivity limits of the assay for detecting limonene, myrcene, γ-terpinene, and β-pinene were determined by comparing the rate of their reaction with DPPH to negative controls containing only DPPH and dodecane. All measurements were performed with n = 3 replicates. It was observed in several instances that data in the first 5 min of the assay were particularly noisy and a stabilisation period was necessary (e.g. Additional file 1F-G). Therefore, a five minute assay window was selected after the stabilisation period, between 7–12 min. The reaction rate within the 7–12 min assay window was calculated by performing a linear regression across these data points. The Pearson product–moment correlation coefficient was calculated for the 7–12 min assay window, and data were rejected where *r* < 0.95.

### Fermentation conditions for limonene biosynthesis with *S. cerevisiae*

Limonene synthases from *Citrus limon* (GenBank AF514287.1) [39] and *Mentha spicata* (GenBank L13459.1) [41,51] were cloned into the expression plasmid pCEV-G2-Ph. Briefly, the plastid targeting sequence was removed from each gene in order to avoid potential misfolding and inclusion body formation [52] and the genes were codon-optimized for expression in yeast. The expression plasmid pCEV-G2-Ph contains a *Saccharomyces* 2mu origin of replication, a phleomycin resistance gene as a selectable marker, and the limonene synthase coding sequence under control of the *S. cerevisiae* transcription elongation factor 1 (TEF1) promoter (Genbank KF154123). Expression plasmids were transformed into *S. cerevisiae* EPY210C, which was generated by curing plasmid pRS425ADS from strain EPY210 (BY4742, PGAL1-tHMGR PGAL1-upc2-1 [pRS425ADS]; [53]). Briefly, *S. cerevisiae* EPY210C contains a truncated, soluble form of 3-hydroxy-3-methylglutaryl-coenzyme A reductase (tHMGR) [54] and *upc2-1*, a global transcription factor involved in upregulation of sterol biosynthesis in *S. cerevisiae*[55]. Both of these features are regulated by a galactose-inducible promoter. *S. cerevisiae* EPY210C transformed with the empty pCEV-G2-Ph vector was used as a negative control in all conditions tested.

Initially, three medium compositions were trialled: complete SD medium prepared according to the manufacturer’s instructions, YP medium [50], and a supplemented YP medium (YP+) to which magnesium sulphate (2 mM) and trace metals and vitamins described by Brennan et al. [20] were added. All media contained 20 μg phleomycin/mL in order to ensure plasmid maintenance. Cells were revived from glycerol stocks by streaking onto either complete SD agar, or YP agar containing 2 g glucose/L [50] and incubating at 30°C. Pre-cultures in either complete SD medium or YP (10 mL in 100 mL baffled Erlenmeyer flasks) were inoculated from single colonies and incubated overnight at 30°C with shaking (200 rpm, 25 mm orbit). Pre-culture media contained 2 g glucose/L.

Fermentation media were the same as pre-culture media except that glucose was replaced with 18 g galactose/L and 2 g glucose/L. Pre-cultures were used to inoculate fermentation media to an OD_660_ of 0.05.

Small scale (5 mL) screening cultures were examined by culturing in a polypropylene 24-well deep-well culture block (QIAGEN cat. no. 19583, QIAGEN, VIC, Australia). Replicate cultures (n = 3) were prepared for each strain and condition. Following inoculation, the 24-well block was sealed with a solvent-resistant foil seal (AlumaSeal film, Excel Scientific, CA., USA) and incubated at 30°C for 120 h with shaking (250 rpm, 25 mm orbit). At the end of the fermentation period, the culture block was chilled at 4°C for 1 h (with the aim of condensing volatile components in the flask head space). The foil seal was removed and 250 μL dodecane (i.e. 5%, v/v) was quickly added to each well before resealing the block with a fresh foil seal. Recovery of hydrophobic compounds from liquid culture in a reduced volume of dodecane imparts an advantage in that the hydrophobic compounds become more concentrated in the smaller volume [56]. The block was shaken for 1 h at room temperature and then chilled at 4°C for 1 h. The dodecane layer was separated by centrifugation at 4500 × *g* for 15 min. The dodecane layer was aspirated, transferred to microcentrifuge tubes, and centrifuged briefly at full speed to facilitate sampling without contamination by the aqueous phase.

Shake-flask fermentations were performed with 50 mL medium in non-baffled 250 mL Erlenmeyer flasks with Teflon-lined screw-cap lids. Dodecane (1 mL) was added at the same time as inoculation and cultures were incubated at 30°C with shaking (200 rpm, 25 mm orbit). At the end of the fermentation period (120 h), flasks were chilled at 4°C for 1 h before collecting the dodecane layer by centrifugation at 4000 × *g*.

Supplemented SD media were as follows: SD + M, SD medium plus trace metals described by Brennan et al. [20] and an additional 2 mM magnesium sulfate; SD(pH), SD medium adjusted to pH 6.3 with sodium hydroxide; SD(pH) + M, SD(pH) medium plus metals described for SD + M; SD(pH) + N, SD(pH) medium plus an additional 10 g ammonium sulfate/L; SD(pH) + MN, SD(pH) medium plus metals and ammonium sulfate described for SD + M and SD(pH) + N media. These media were selected on the basis that metals, starting pH and nitrogen content were among the largest differences between SD and YP + media (see Additional file 2). All supplemented SD media contained 20 μg phleomycin/mL in order to ensure plasmid maintenance. Pre-culture media contained 2 g glucose/L, while fermentation media were the same as pre-culture media except that glucose was replaced with 18 g galactose/L and 2 g glucose/L. Pre-cultures were used to inoculate fermentation media to an OD_660_ of 0.05.

### GC-MS sample preparation and analysis for limonene production

The high boiling point of dodecane relative to limonene made it unsuitable as a solvent in our GC-MS method. Therefore samples were diluted 100-fold in another solvent prior to injection. Hexane, a 1:4 mixture of ethyl acetate:hexane, and 1:4 toluene:hexane were trialled as dilution solvents. When hexane and 1:4 ethyl acetate:hexane were used, dodecane continued to exert a strong reverse solvent effect [57,58] which caused extensive tailing of limonene peaks. Use of 1:4 toluene:hexane as the diluent resolved this problem and produced uniform peak shapes (Additional file 3). For analysis, dodecane overlay samples were diluted 1 in 100 in 1:4 toluene:hexane. Myrcene was used as an internal standard and was added to the dodecane overlay samples immediately prior to dilution such that the concentration prior to injection was 10 μM. GC-MS was performed at Metabolomics Australia (Queensland Node). Samples (3 μL) were injected in splitless mode at 220°C using helium as carrier gas with a constant flow rate of 2 mL/min. Compounds were separated using a Varian factorFOUR capillary column (VF-5 ms: 0.25 mm internal diameter, 0.25 μm film, 30 m length with a 10 m fused guard column) (Varian, Mulgrave, VIC, Australia) on an Agilent 7890A gas chromatograph connected to an Agilent 5975C MSD mass spectrometer (Agilent, Mulgrave, VIC, Australia). The initial oven temperature was held at 70°C for 10 min, then increased to 300°C at a rate of 40°C/min and held at 300°C for 3 min. The transfer line, ion source, and quadrupole were maintained at 290°C, 300°C, and 150°C, respectively. Analytes were detected in selected ion monitoring mode. Between 4.5 and 6.7 min, characteristic ions for myrcene were monitored with mass to charge (*m*/*z*) ratios of 69.1, 93.1, and 136.5. After 6.7 min, characteristic ions for limonene (*m*/*z* 68.1, 93.1, and 136.5) were monitored. Dwell time for each ion was 5 ms. Analytes were identified by comparison to authentic standards and linear standard curves were obtained for myrcene and limonene concentrations between 0.5 μM and 50 μM. The lower limits of detection were 0.25 μM myrcene and 0.1 μM limonene. All samples for analysis were prepared with n = 3 replicate dilutions from the original sample.

## Abbreviations

YP+: Supplemented YP medium; CLLS: *Citrus limon* limonene synthase; MSLS: *Mentha spicata* limonene synthase; GPP: Geranyl diphosphate; GC-MS: Gas chromatography–mass spectrometry; DPPH: 2,2-diphenyl-1-picrylhydrazyl; SD: Synthetic dextrose; LB: Lysogeny broth.

## Competing interests

The authors declare that they have no competing interests.

## Authors’ contributions

JBYHB conceived of the study, participated in experimental design, executed the experiments, and drafted the manuscript. PC developed the GC-MS methods. CEV and LKN participated in experimental design and helped to draft the manuscript. All authors read and approved the final manuscript.

## Supplementary Material

Additional file 1 **Spectral properties of DPPH dissolved in dodecane and assay optimization.** The absorbance properties of DPPH dissolved in dodecane were examined in order to determine an appropriate DPPH concentration and absorbance wavelength for the assay. The effect of mixing on assay reproducibility was examined, as were the effects of microplate composition, sample aeration, and incubation with live culture on the background reaction rate. Click here for file

Additional file 2 **Culture media components.** Components of YP base medium and SD medium are tabulated, along with additives used in variations trialled in this work. Click here for file

Additional file 3 **Solvent optimization to minimize the reverse solvent effect in dodecane-extracted samples.** Dodecane exerted a strong reverse solvent effect on limonene analytes, preventing quantification. This was resolved with solvent optimization. Click here for file
