# Supplementary material for: 2,2-Diphenyl-1-picrylhydrazyl as a screening tool for recombinant monoterpene biosynthesis
Source: Microb Cell Fact. 2013 Aug 23;12:76. doi: 10.1186/1475-2859-12-76 (PMC3847554; doi:10.1186/1475-2859-12-76)
Supplement: Additional file 1 — Spectral properties of DPPH dissolved in dodecane and assay optimization. The absorbance properties of DPPH dissolved in dodecane were examined in order to determine an appropriate DPPH concentration and absorbance wavelength for the assay. The effect of mixing on assay reproducibility was examined, as were the effects of microplate composition, sample aeration, and incubation with live culture on the background reaction rate. [file 1475-2859-12-76-S1.docx]

**Additional file 1: Assay optimization**

**Spectral properties of DPPH dissolved in dodecane**

**
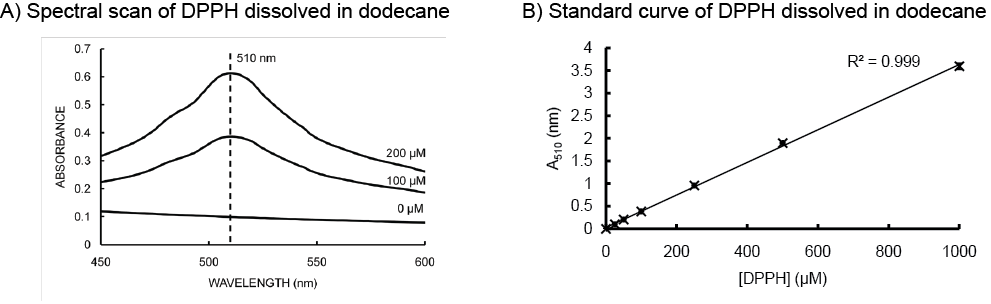
**

A) Spectral scan of 0, 100, and 200 µM DPPH dissolved in dodecane, measuring the absorbance every 2 nm. The peak absorbance of 510 nm is similar to the reported value for DPPH dissolved in methanol (517 nm), and the broadness of the peak suggests that either value would be acceptable.

B) Standard curve of DPPH dissolved in dodecane (n = 3, mean ± 1 SD).

**Factors influencing the background reaction rate of DPPH dissolved in dodecane.**
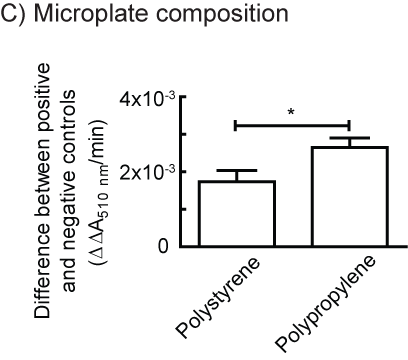

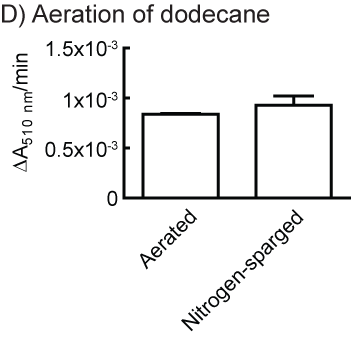

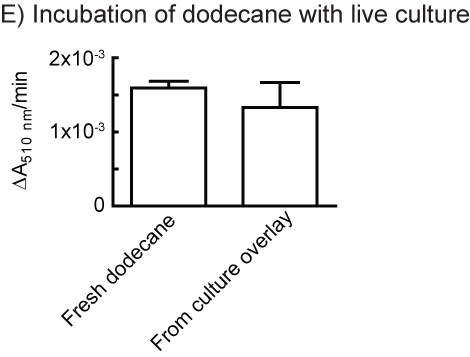


C) The reaction rates (ΔA510 nm/min) of 100 µM DPPH with 0 µM or 500 µM limonene in polystyrene and polypropylene 96-well microtitre plates were measured. The difference in rate between 0 µM and 500 µM limonene was calculated and compared between polystyrene and polypropylene plates using an unpaired Student’s *t*-test (n = 3, mean ± SD, * = p < 0.05). Reaction rates of DPPH with dodecane were also compared when dodecane had been aerated or treated with nitrogen (D) and after incubation with live *S. cerevisiae* EPY210C culture (E) (n = 3, mean ± SD).

**Importance of plate mixing**

**
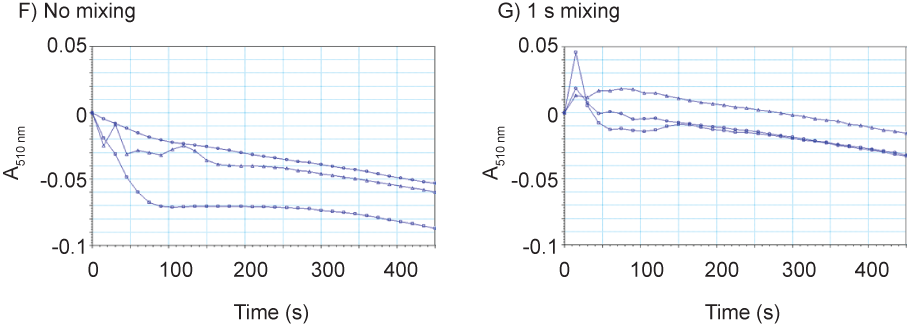
**

DPPH (final concentration 100 µM) was added to limonene (final concentration 250 µM) and absorbance at 510 nm was monitored either immediately (F) or after 1 s mixing using the automix function of the plate reader (G). The raw data from the Spectramax M5 software (Softmax Pro 5.3, Molecular Devices, CA) are shown to give an example of typical experimental output. Three replicates are shown in each instance with measurements (○,△,◊) with measurements recorded every 30 s. Mixing improved the reproducibility of the reaction curves.
