# Supplementary material for: 2,2-Diphenyl-1-picrylhydrazyl as a screening tool for recombinant monoterpene biosynthesis
Source: Microb Cell Fact. 2013 Aug 23;12:76. doi: 10.1186/1475-2859-12-76 (PMC3847554; doi:10.1186/1475-2859-12-76)
Supplement: Additional file 2 — Culture media components. Components of YP base medium and SD medium are tabulated, along with additives used in variations trialled in this work. [file 1475-2859-12-76-S2.docx]

**Additional file 2: Culture media components**

In instances where the pH of SD medium was modified, sodium hydroxide was used to adjust the starting pH to 6.3.

| **YP base** |  | |  | **SD (MP Biomedicals)** | | |  | **Additives for YP+ and SD medium variants** | | |
| --- | --- | --- | --- | --- | --- | --- | --- | --- | --- | --- |
| **Compound** | **Final Conc** | |  | **Compound** | **Final Conc** | |  | **Metals** | **Final Conc** | |
| Total N | 3.31 | g L^-1^ |  | (NH_4_)_2_SO_4_ | 5 | g L^-1^ |  | ZnSO_4_·7H_2_O | 4.5 | mg L-1 |
| Free NH3N | 38.6 | mg L^-1^ |  | KH_2_PO_4_ | 1 | g L^-1^ |  | EDTA | 15 | mg L-1 |
| Phosphorous | 14 | mg L^-1^ |  | NaCl | 0.1 | g L^-1^ |  | MnCl_2_·2H_2_O | 0.84 | mg L-1 |
| NaCl | 0.42 | g L^-1^ |  | CaCl_2_ | 0.1 | g L^-1^ |  | CoCl_2_·6H_2_O | 0.3 | mg L-1 |
| Mg^2+^ | 0.2 | mg L^-1^ |  | MgSO_4_ | 0.5 | g L^-1^ |  | CuSO_4_·5H_2_O | 0.3 | mg L-1 |
| **Vitamins** |  |  |  | **Vitamins** |  |  |  | Na_2_MoO_4_·2H_2_O | 0.4 | mg L-1 |
| Niacin | 1.2 | mg L^-1^ |  | Biotin | 2 | µg L^-1^ |  | CaCl2·2H2O | 4.5 | mg L-1 |
| Riboflavin | 0.2 | mg L^-1^ |  | Calcium panthothenate | 0.4 | mg L-1 |  | FeSO4·7H2O | 3 | mg L-1 |
| **Trace metals** |  |  |  | Niacin | 0.4 | mg L-1 |  | H3BO3 | 1 | mg L-1 |
| Zinc | 0.2 | mg L^-1^ |  | Inositol | 2 | mg L-1 |  | KI | 0.1 | mg L-1 |
| Copper | 0.2 | mg L^-1^ |  | Thiamine HCl | 0.4 | mg L-1 |  |  |  |  |
| Iron | 1.2 | mg L^-1^ |  | Pyridoxine HCl | 0.4 | mg L-1 |  | **Vitamins for YP+** | **Final Conc** | |
| Calcium | 4.96 | mg L^-1^ |  | para-aminobenzoic acid | 0.2 | mg L-1 |  | D-biotin | 0.05 | mg L-1 |
| Cobalt | 3.4 | mg L^-1^ |  | Folic Acid | 2 | µg L^-1^ |  | Calcium panthothenate | 1 | mg L-1 |
| **Amino Acids** |  |  |  | Riboflavin | 0.2 | mg L-1 |  | Nicotinic acid | 1 | mg L-1 |
| Cystine | 20 | mg L^-1^ |  | **Trace metals** |  |  |  | Myoinositol | 1 | mg L-1 |
| Arginine | 1.6 | g L^-1^ |  | ZnSO_4_ | 0.4 | mg L^-1^ |  | Thiamine HCl | 25 | mg L-1 |
| Glutamic Acid | 1.82 | g L^-1^ |  | MnSO_4_ | 0.4 | mg L^-1^ |  | Pyridoxal HCl | 1 | mg L-1 |
| Histidine | 0.18 | g L^-1^ |  | CuSO_4_ | 0 | mg L^-1^ |  | para-aminobenzoic acid | 0.2 | mg L-1 |
| Isoleucine | 0.32 | g L^-1^ |  | FeCl_3_ | 0.2 | mg L^-1^ |  |  |  |  |
| Leucine | 0.56 | g L^-1^ |  | Na_2_MoO_4_ | 0.2 | mg L^-1^ |  | **Additional nitrogen** | **Final Conc** | |
| Lysine | 0.86 | g L^-1^ |  | H_3_BO_4_ | 0.5 | mg L^-1^ |  | (NH_4_)_2_SO_4_ | 10 | g L^-1^ |
| Methionine | 0.2 | g L^-1^ |  | KI | 0.1 | mg L^-1^ |  |  |  |  |
| Phenylalanine | 0.38 | g L^-1^ |  | **Amino Acids** |  |  |  |  |  |  |
| Threonine | 0.36 | g L^-1^ |  | Adenine | 10 | mg L^-1^ |  |  |  |  |
| Tryptophan | 40 | mg L^-1^ |  | L-Arginine HCl | 50 | mg L^-1^ |  |  |  |  |
| Tyrosine | 0.16 | g L^-1^ |  | L-Aspartic Acid | 80 | mg L^-1^ |  |  |  |  |
| Valine | 0.44 | g L^-1^ |  | L-Histidine HCl | 20 | mg L^-1^ |  |  |  |  |
| Proline | 2.8 | g L^-1^ |  | L-Isoleucine | 50 | mg L^-1^ |  |  |  |  |
|  |  |  |  | L-Leucine | 100 | mg L^-1^ |  |  |  |  |
|  |  |  |  | L-Lysine HCl | 50 | mg L^-1^ |  |  |  |  |
|  |  |  |  | L-Methionine | 20 | mg L^-1^ |  |  |  |  |
|  |  |  |  | L-Phenylalanine | 50 | mg L^-1^ |  |  |  |  |
|  |  |  |  | L-Threonine | 100 | mg L^-1^ |  |  |  |  |
|  |  |  |  | L-Tryptophan | 50 | mg L^-1^ |  |  |  |  |
|  |  |  |  | L-Tyrosine | 50 | mg L^-1^ |  |  |  |  |
|  |  |  |  | Uracil | 20 | mg L^-1^ |  |  |  |  |
|  |  |  |  | L-Valine | 140 | mg L^-1^ |  |  |  |  |
