# Supplementary material for: 2,2-Diphenyl-1-picrylhydrazyl as a screening tool for recombinant monoterpene biosynthesis
Source: Microb Cell Fact. 2013 Aug 23;12:76. doi: 10.1186/1475-2859-12-76 (PMC3847554; doi:10.1186/1475-2859-12-76)
Supplement: Additional file 3 — Solvent optimization to minimize the reverse solvent effect in dodecane-extracted samples. Dodecane exerted a strong reverse solvent effect on limonene analytes, preventing quantification. This was resolved with solvent optimization. [file 1475-2859-12-76-S3.docx]

**Additional file 3**

**Development of a GC-MS method for analysing low concentrations of limonene extracted in dodecane**

A) 5 µM limonene in hexane (diluted from 500 µM limonene dissolved in dodecane)


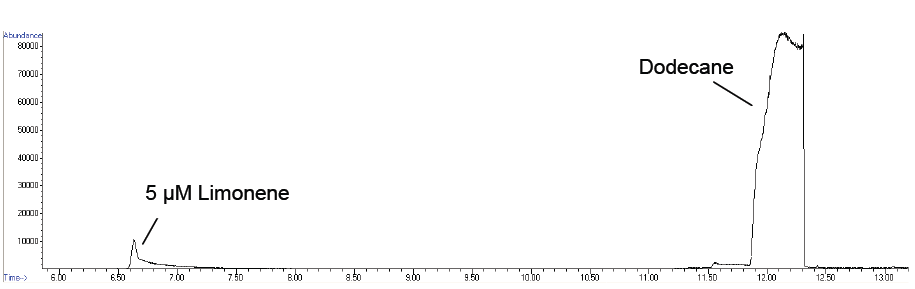


Extensive peak tailing was observed when a 500 µM solution of limonene was diluted to 5 µM in hexane, preventing quantification of the peak area. This is due to the reverse solvent effect exerted by the high concentration of dodecane that remains in the diluted sample. The reverse solvent effect is the physical displacement of an analyte in the chromatography column by an extremely abundant analyte (or bulk solvent) that has a later retention time than the analyte of interest [[47-49](#_ENREF_47)]. The analyte peak is therefore spread over a larger area because of this forward displacement.

B) 5 µM limonene in 1:4 toluene:hexane (diluted from 500 µM limonene dissolved in dodecane)
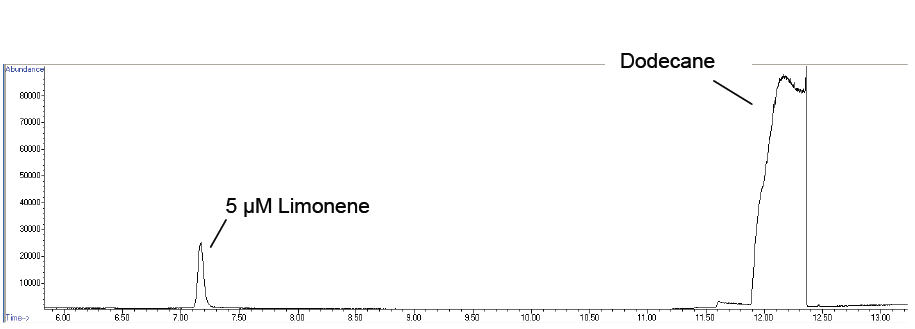


The reverse solvent effect was resolved by changing the dilution solvent to a 1:4 mixture of toluene:hexane. The boiling point of toluene (111 °C) is between the boiling points of hexane (68 °C) and limonene (176 °C). The introduction of a bulk volume of toluene into the sample may physically displace the limonene peak in the opposite direction to dodecane. Use of 1:4 toluene:hexane for dilution of the sample adequately compressed the limonene peak for integration.
